# Erythropoietin Attenuates Experimental Contrast-Induced Nephrology: A Role for the Janus Kinase 2/Signal Transducer and Activator of Transcription 3 Signaling Pathway

**DOI:** 10.3389/fmed.2021.634882

**Published:** 2021-04-13

**Authors:** Jia Yang, Jiaojiao Zhou, Xin Wang, Ling Ji, Siwen Wang, Xuelian Chen, Lichuan Yang

**Affiliations:** ^1^Division of Nephrology, Department of Medicine, West China Hospital of Sichuan University, Chengdu, China; ^2^Division of Ultrasound, West China Hospital of Sichuan University, Chengdu, China; ^3^Department of Pediatric Nephrology, West China Second University Hospital, Sichuan University, Chengdu, China

**Keywords:** erythropietin, contrast-induced nephrology, JAK2, stat3, apoptosis

## Abstract

The aim of the present study was to investigate the effect of erythropoietin (EPO) on contrast-induced nephrology (CIN) *in vivo* and *in vitro*. Male C57BL/6J mice were divided into four groups: control, CIN (iohexol 6.0 g/kg), EPO (3,000 IU/kg), and CIN+EPO. Hematoxylin and eosin (H&E) staining and biochemical index analyses were performed to evaluate renal injury. The cellular proliferation rate was detected using the Cell Counting Kit-8 (CCK-8) assay. In addition, a terminal deoxynucleotidyl transferase-mediated dUTP nick end labeling (TUNEL) assay and flow cytometric assay were used to assess the apoptosis of tissue and cells, respectively. Renal protein expression associated with apoptosis, pyroptosis, and signaling pathways was determined by Western blot (WB) assays for tissues and cells. The results showed that EPO significantly decreased serum creatinine, blood urea nitrogen, and cystatin C levels and alleviated renal histological changes *in vivo*. The protein levels of Janus kinase 2/signal transducer and activator of transcription 3 (JAK2/STAT3) signaling pathway components were overexpressed in the EPO treatment group. Furthermore, EPO suppressed the cell apoptosis and pyroptosis; decreased the protein levels of cleaved caspase-3, Bax, gasdermin D (GSDMD), and caspase-1; and enhanced the expression of Bcl-2. In summary, EPO could exert renoprotective effect by activating the JAK2/STAT3 signaling pathway, which may be a novel potential therapy for the treatment of CIN in the clinic.

## Introduction

Contrast-induced nephrology (CIN), as the name indicates, is an acute kidney injury (AKI) caused by endovascular application of contrast medium (CM) when excluding other causative factors. CIN has been discovered as the third most common cause of hospital-acquired AKI (1). The incidence of CIN ranges from 10 to 30% according to different studied populations and diagnostic criteria (1–4). The underlying mechanisms of CIN are complicated and not yet completely expounded. Current studies suggest that the occurrence of CIN is mainly associated with the following mechanisms: hypoxia damage to the renal parenchyma, direct toxicity of contrast media, oxidative stress, immunizing inflammatory response, cell pyroptosis, and apoptosis (5, 6).

Erythropoietin (EPO) is an endogenous cell factor excreted by the kidney and is regarded initially as a hematopoietic factor. Recombinant human EPO has played a part in the treatment of renal anemia in patients with end-stage renal disease (ESRD) or chronic kidney disease. More recently, the protective effect of EPO on multiple tissues, including the kidney, has been reported (7–9).

The Janus kinase (JAK)/signal transducer and activator of transcription (STAT) signaling pathway, as an essential downstream mediator of a variety of metabolically relevant hormones, including EPO, is one of the major signaling pathways of cell metabolism and apoptosis (10). The JAK family includes JAK1/2/3 and TYK2, and the STAT family consists of STAT1/2/3/4, STAT5A/B, and STAT6, which have been confirmed in mammals. JAK1/2 and STAT3/5/6 are involved in the process of AKI and have been shown in experimental AKI or ischemia–reperfusion injury models (11–13).

Therefore, we hypothesized that EPO could also attenuate renal injury caused by CM. In this study, we provide evidence that systemically administered EPO could attenuate CIN by enhancing the JAK2/STAT3 signaling pathway.

## Materials and Methods

### Animals and Contrast-Induced Acute Kidney Injury Model

The Animal Experimentation Ethics Committees of West China Medical Center and Institutes of Animal Science approved this study. Healthy C57BL/6J mice (male, 8–10 weeks old, weighing 20–26 g) were purchased from the Experimental Animal Center at the West China Medical Center of Sichuan University; housed in a specific pathogen-free, temperature-controlled environment; and kept on a 12-h light/dark cycle with standardized feeding and drinking. The experimental mice were randomly divided into four groups (*n* = 18 in each group) as follows: control group, EPO group, CIN group, and EPO+CIN group. The mouse model of CIN was a modified version of a previously described model (14, 15). In brief, after 7 days of acclimation to the experimental area and 24 h of water deprivation, mice were injected with indomethacin (10 mg/kg; MedChemExpress, NJ, USA) and *N*^G^-nitro-l-arginine methyl ester (10 mg/kg; MedChemExpress, NJ, USA) intraperitoneally before iohexol (6.0 g/kg organically bound iodine, General Pharmaceutical Co., Ltd., Shanghai, China). Normal saline was injected at each time point in the control group. To explore the possible protection of EPO against CIN, recombinant human EPO (3,000 IU/kg) or saline was administered subcutaneously 1 h before the indomethacin injection. At 6, 24, and 48 h after CM injection, the animals were euthanized using a 10% chloral hydrate to collect blood and kidney samples for various examinations.

### Renal Function

Serum creatinine (Scr), blood urea nitrogen (BUN), and cystatin C (CysC), serving as indicators of kidney function, were measured by enzymatic methods using the respective assay kits (Mindray, Shenzhen, China).

### Kidney Histology

After fixation in 10% formalin for 48 h, kidneys were dehydrated in a graded ethyl alcohol series, embedded in paraffin, and then cut into 4-μm sections. Tissue sections were subjected to hematoxylin and eosin (H&E) staining for morphologic analysis. H&E-stained tissue slices were observed by optical microscopy at ×200 magnification, and 10 different fields of renal interstitium from each group were randomly selected for semiquantitative analysis. According to a previous study (16), the semiquantitative scoring criteria of renal tubular histopathology focused on tubular cell swelling and vacuolation were as follows: 0, no abnormalities; 1+, changes affecting <25% of the sample; 2+, changes affecting 25–50%; 3+, changes affecting 50–75%; and 4+, changes affecting more than 75%.

### Terminal Deoxynucleotidyl Transferase-Mediated dUTP Nick End Labeling Assay

Terminal deoxynucleotidyl transferase-mediated dUTP nick end labeling (TUNEL) staining was performed to detect cell apoptosis, and sections were stained using the DeadEnd™ Fluorometric Apoptosis Detection Kit (Promega, Madison, WI, USA) according to the manufacturer's instructions. The number of TUNEL-positive tubular cells was counted under an upright fluorescence microscope at ×400 magnification.

### Cell Culture and Treatment

Human kidney proximal tubular epithelial cells (HK-2 cells) were obtained from American Type Culture Collection (Manassas, VA, USA) and cultured in DMEM/F-12 medium supplemented with 10% fetal bovine serum. Cells were maintained in a humidified incubator at 37°C (5% CO_2_). Cells were pretreated with different concentrations (25, 50, and 100 IU/ml) of EPO for 1 h and then treated with iohexol (75 mgI/ml) to mimic CIN *in vitro*. Cells maintained in normal medium were used as controls.

### Annexin V/Propidium Iodide Assay

The apoptosis rate was analyzed using an Annexin V-FITC/propidium iodide (PI) apoptosis detection kit (4A Biotech Co. Ltd, Beijing, China). Briefly, cells were seeded into 6-well plates (1.5 × 10^5^ cells/well) and cultured in medium with free serum for 24 h. After treatment with EPO (100 IU/ml) and iohexol (75 mgI/ml), cells were harvested and washed twice with phosphate-buffered saline (PBS) and then resuspended in binding buffer mixed with Annexin V-FITC reagent and PI reagent according to the manufacturer's protocol. Apoptotic cells were measured by flow cytometry (Olympus, IX71).

### Cell Counting Kit-8 Assay

Cell viability was determined by a Cell Counting Kit-8 (CCK-8) assay kit (Us Everbright Inc., CA, USA) according to the manufacturer's protocol. HK-2 cells were seeded into 96-well plates at a density of 1.0 × 10^4^ cells/well. After treatment with or without EPO and iohexol, cells were incubated with 10 μl of CCK-8 solution in each well at 37°C for 2 h. Then, we measured the absorbance at 450 nm and calculated the optical density.

### Western Blotting

Total proteins of cultured HK-2 cells and kidneys of the mice were extracted with cell lysis buffer supplemented with protease and phosphatase inhibitor cocktails. The concentration of proteins was detected with a BCA Protein Assay Kit (Thermo Fisher Scientific, Waltham, MA, USA). Equal amounts of proteins were separated by 10% sodium dodecyl sulfate–polyacrylamide gel electrophoresis (SDS-PAGE) and transferred to polyvinylidene fluoride membranes. After being blocked with 5% skim milk for 1 h, the membranes were incubated overnight at 4°C with antibodies directed against total STAT3 and phospho-STAT3-Tyr705 (1:2,000, Cell Signaling Technology), total JAK2 (1:1,000, Cell Signaling Technology), phospho-JAK2-Tyr1007/1008 (1:2,000, Abcam), cleaved caspase-3 (1:1,000, Cell Signaling Technology), Bax (1:2,000, ProteinTech), Bcl-2(1:1,000, ProteinTech), gasdermin D (GSDMD, 1:1,000, Abcam), and caspase-1 (1:2,000, Huabio). The next day, the membranes were washed and incubated with horseradish peroxidase-conjugated secondary antibodies for 1 h at room temperature. Protein bands were visualized using enhanced chemiluminescence substrate and quantified by ImageJ software ver. 1.8.

### Statistical Analysis

All data are presented as the means ± standard deviations (SDs). Differences between the groups were determined using one-way ANOVA. Statistical analysis was performed using GraphPad Prism 7.0 software (GraphPad Software, La Jolla, CA, USA). The results were recognized as statistically significant when *P* < 0.05.

## Result

### Erythropoietin Ameliorates Renal Dysfunction and Tissue Damage in Contrast-Induced Nephrology

**Figure 1:**
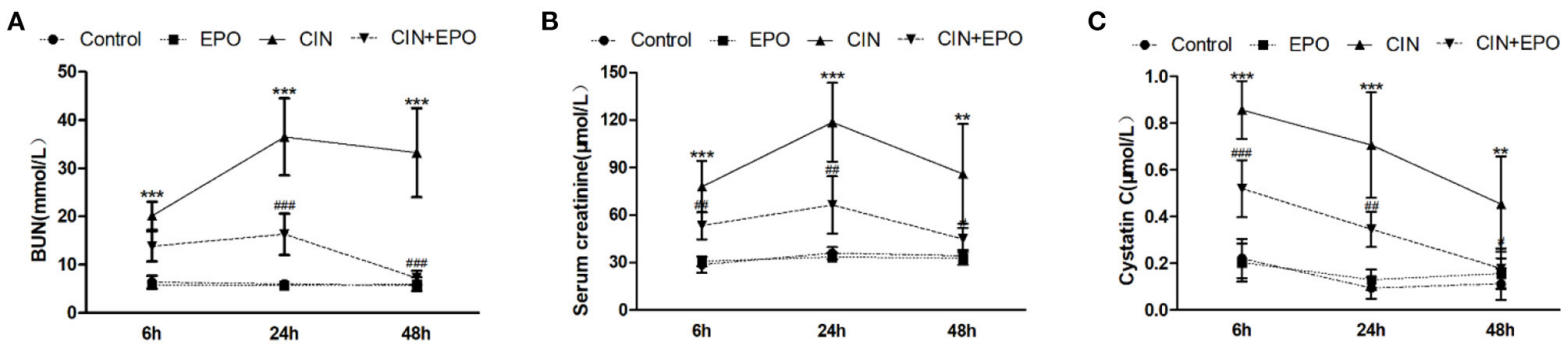
Effect of EPO administration in renal functional parameters during CIN. **(A)** BUN (mmol/L), **(B)** serum creatinine (μmol/L), and **(C)** cystatin C (μmol/L). Values are the mean ± SD (*n* = 6 mice/each group). **P < 0.01 and ***P < 0.001 vs. the control groups; ^#^*P* < 0.05, ^*##*^*P* < 0.01, and ^*###*^*P* < 0.001 vs. the CIN groups. BUN, blood urea nitrogen; EPO, erythropoietin; CIN, contrast-induced nephrology.

The CIN model was confirmed by the elevation of biochemical parameters of renal dysfunction and histopathology evaluation. As shown in Figure 1A, serum BUN was significantly increased in the CIN group at all time points compared with the control group and reached maximum levels at 24 h in the study. Moreover, compared with the control group, the levels of Scr (Figure 1B) and cystatin C (Figure 1C) were dramatically increased, reaching maximum levels at 24 and 6 h, respectively. High levels of BUN, Scr, and CysC caused by CM were obviously reduced by pretreatment with EPO.

### Erythropoietin Reduces Histopathological Changes in the Kidneys of Contrast-Induced Nephrology Mice

**Figure 2:**
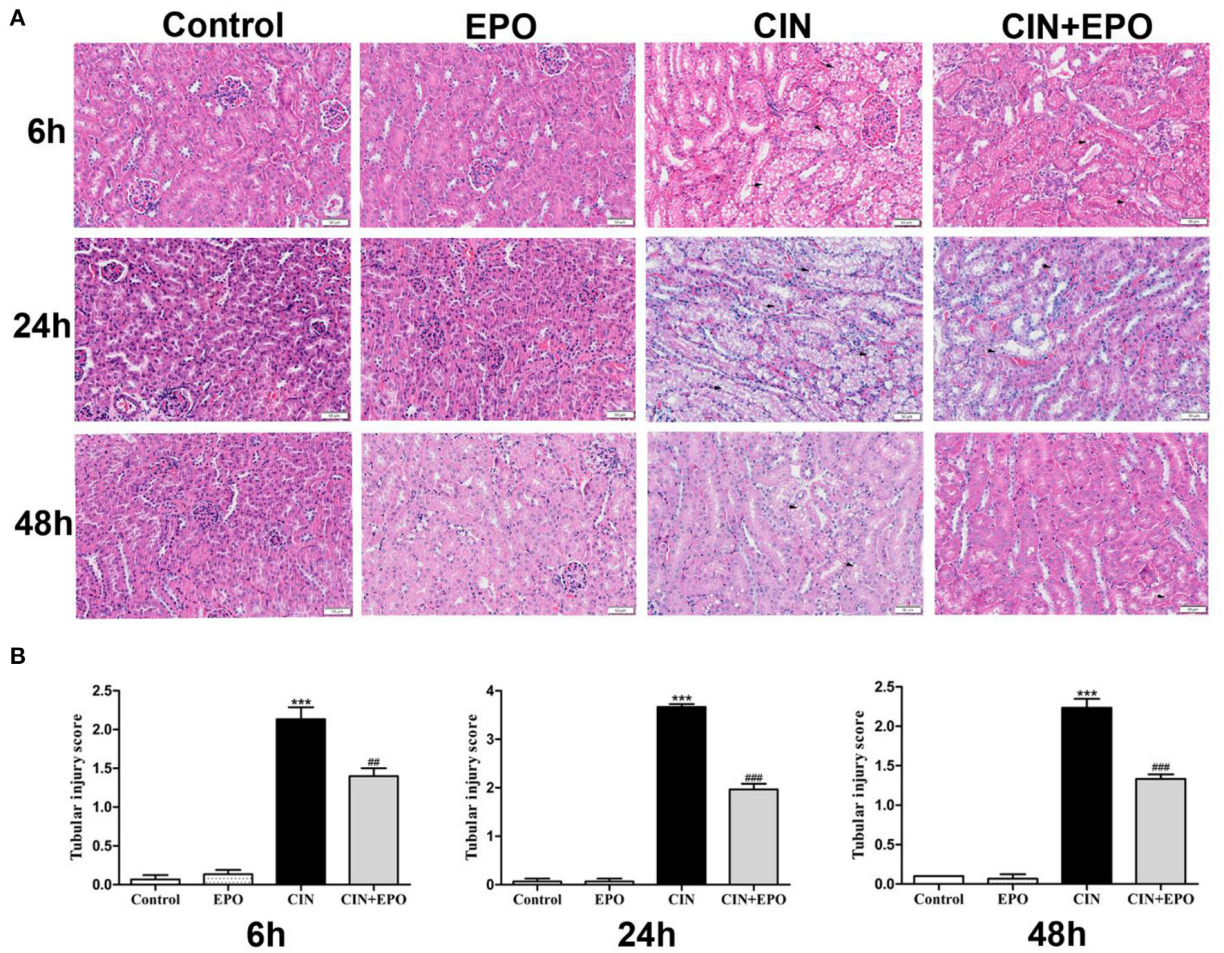
Effect of EPO administration in tubular injury score in CIN. **(A)** In the four groups, representative images of kidney tissues were stained with H&E at various time points (magnification ×200). **(B)** The average renal tubular injury scores in the four groups at various time points. ***P < 0.001 vs. the control groups. ^*##*^*P* < 0.01, ^*###*^*P* < 0.001 vs. the CIN groups. EPO, erythropoietin; CIN, contrast-induced nephrology.

Renal histological changes were assessed using H&E staining. As shown in Figure 2A, extensive tubular injury, characterized by tubular cell swelling and vacuolation, was observed in the CIN group and reached a peak at 24 h. Compared with the CIN group, the average renal tubular injury scores in the CIN+EPO group were significantly ameliorated across time points (Figure 2B).

### Erythropoietin Alleviates Apoptosis in Renal Contrast-Induced Nephrology

**Figure 3:**
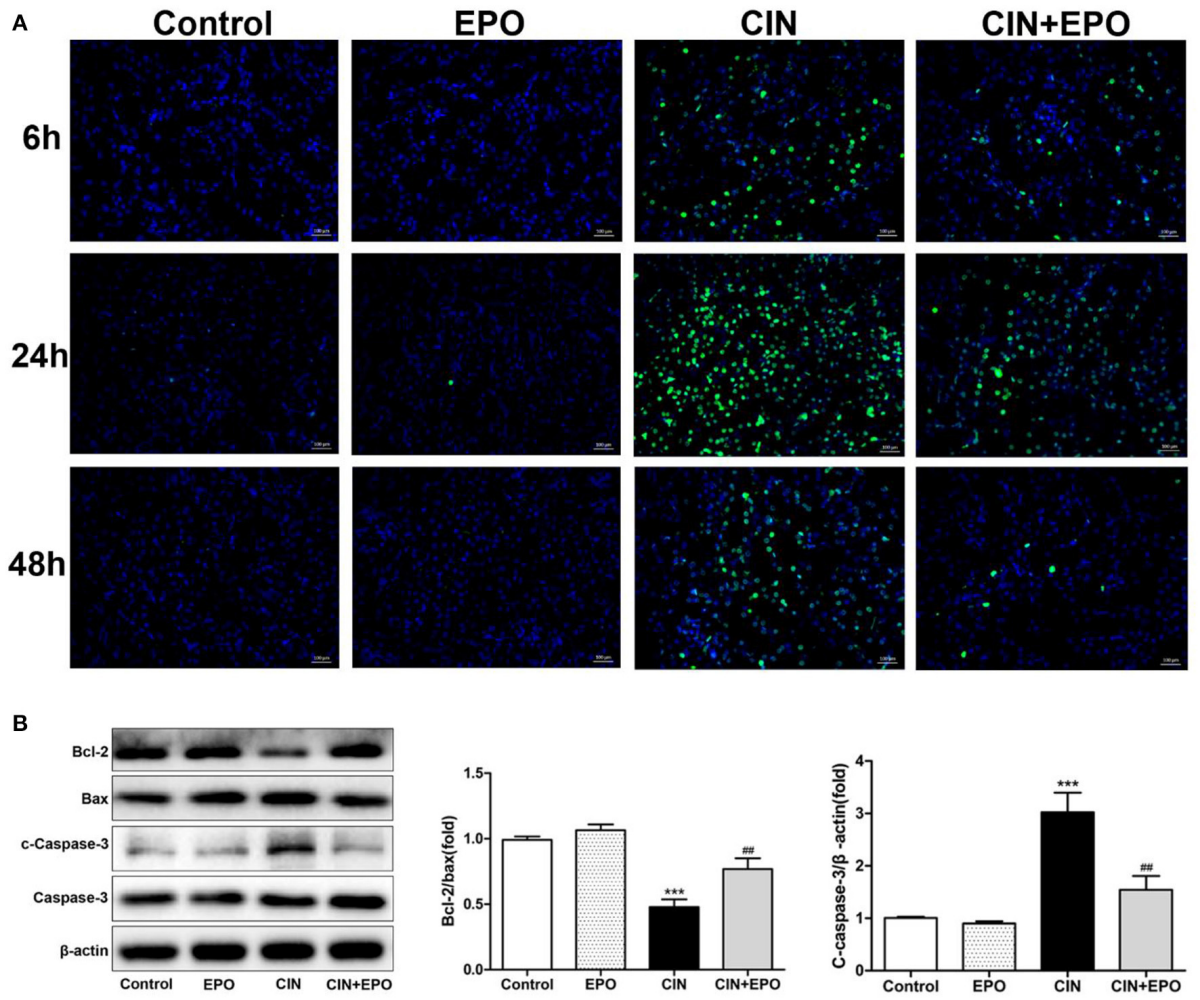
Effect of EPO on apoptosis of renal tissue in CIN. **(A)** TUNEL staining was used to assess the kidney cell apoptosis in the presence and absence of EPO. Original magnification, × 400. **(B)** Apoptosis-related proteins, including Bax, cleaved caspase-3, and Bcl-2, were determined by Western blot assay. Each bar represents the mean ± SD calculated from three independent experiments. ***P < 0.001 vs. the control groups; ^*##*^*P* < 0.01 vs. the CIN groups. EPO, erythropoietin; CIN, contrast-induced nephrology; TUNEL, terminal deoxynucleotidyl transferase-mediated dUTP nick end labeling.

Emerging evidence has demonstrated that apoptosis is involved in the development of CIN. Our results from the TUNEL assay are shown in Figure 3A. The CIN group showed a significant increase in the apoptosis rate, which was decreased by EPO pretreatment. The peak values of cell apoptosis by observation of green-stained cells in the field of vision were also observed at 24 h. In addition, the production of apoptosis-related proteins was determined by Western blot (WB) assay at 24 h post-CM. The results revealed that the levels of Bax and cleaved caspase-3 significantly decreased while the levels of Bcl-2 obviously increased in the CIN group, and they returned with the administration of EPO (Figure 3B).

### Erythropoietin Preconditioning Alleviates the Inhibition of HK-2 Cell Proliferation Induced by Contrast Medium

**Figure 4:**
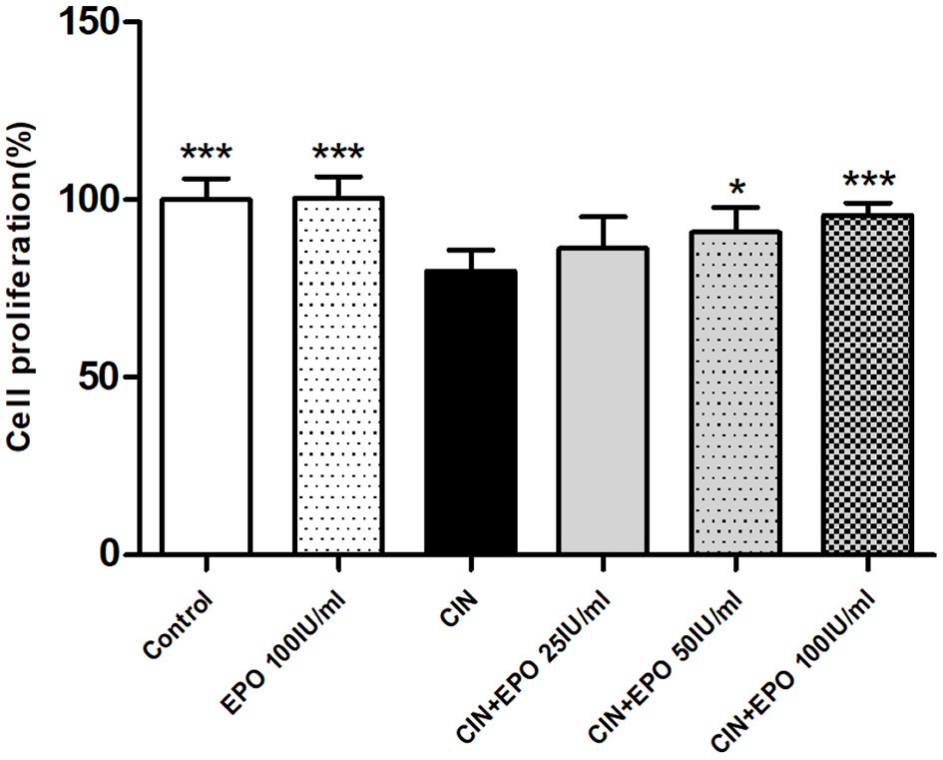
Change of proliferation rate in each group. Cell Counting Kit-8 assay was used to determine the proliferation rate. Data are expressed as mean ± S.D. *P < 0.05, ***P < 0.001 vs. the CIN group. EPO, erythropoietin; CIN, contrast-induced nephrology.

As shown in Figure 4, after a 24-h incubation period with CM, the proliferation rate of the CIN group was much lower than that of the control group. With pretreatment with EPO at a concentration of 50 or 100 IU/ml, especially the latter, the proliferation rate was found to be higher than that in the CIN group, while 25 IU/ml EPO failed to enhance the proliferation rate.

### Erythropoietin Inhibited Apoptosis and Pyroptosis of Tubular Epithelial Cells in Response to Contrast Medium

**Figure 5:**
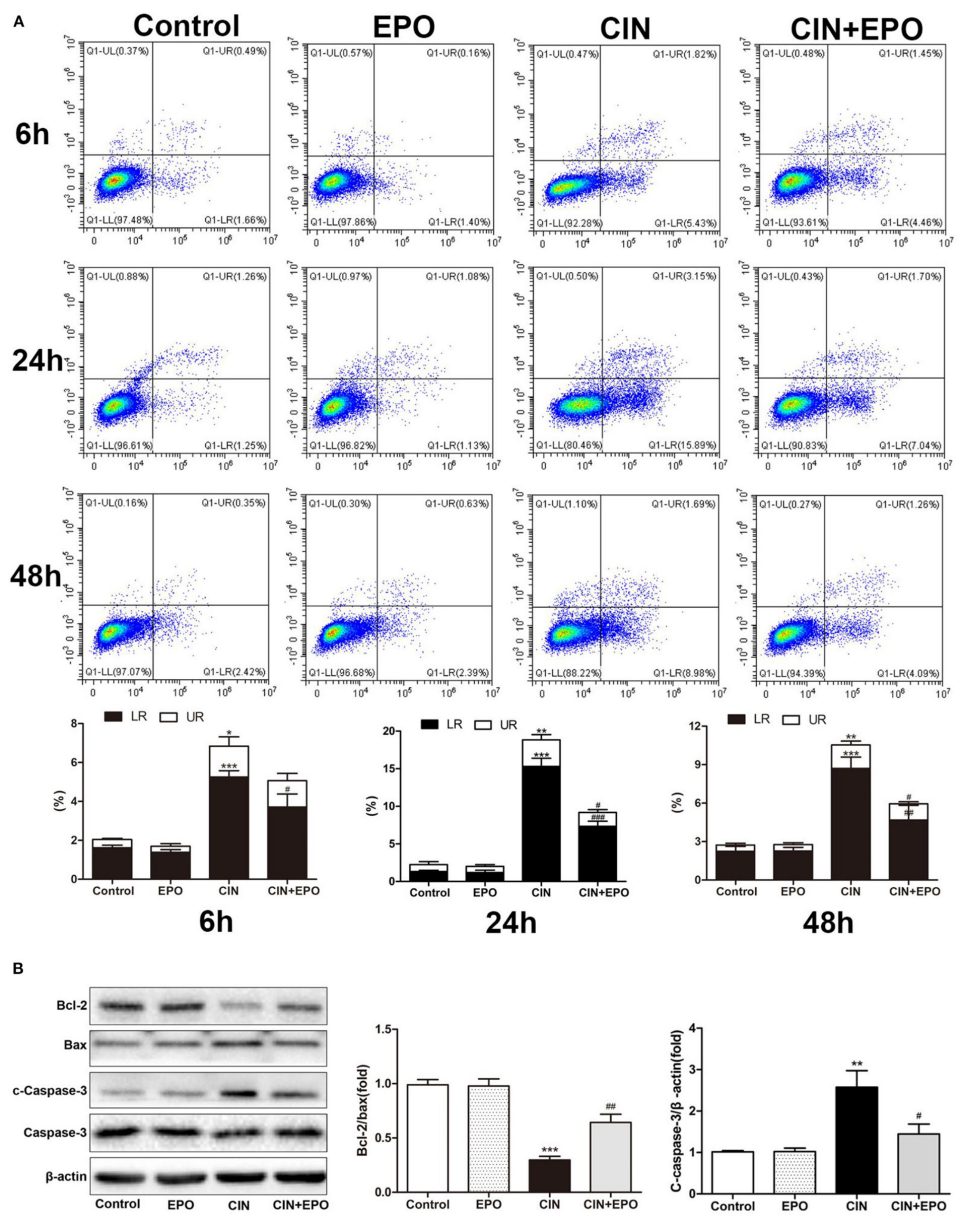
Effect of EPO on apoptosis induced by contrast in HK-2 cells. **(A)** Annexin V/PI assay was used to assess the cell apoptosis and pyroptosis in the presence and absence of EPO. **(B)** Apoptosis-related proteins, including Bax, cleaved caspase-3, and Bcl-2, were determined by Western blot assay. Each bar represents the mean ± SD calculated from three independent experiments. *P < 0.05, **P < 0.01, ***P < 0.001 vs. the control group; ^#^*P* < 0.05, ^*##*^*P* < 0.01, ^*###*^*P* < 0.001 vs. the CIN group. EPO, erythropoietin; CIN, contrast-induced nephrology; PI, propidium iodide.

Cell apoptosis and pyroptosis rate were evaluated by Annexin V-FITC/PI apoptosis detection. Apoptosis protein levels were detected by WB. The results of cell apoptosis and pyroptosis rate are shown in Figure 5A. In contrast to the control group, the CIN group's apoptotic and pyroptosis rates increased at 6 h, peaked at 24 h, and then gradually decreased. Pretreatment with EPO attenuated early cell apoptosis at all time points and inhibited late cell apoptosis and pyroptosis at any other time point except 6 h. The expression levels of apoptosis proteins at 24 h post-CM were in accord with the corresponding apoptotic rate, while Bcl-2, an anti-apoptotic effector, was decreased in the CIN group and recovered with EPO treatment (Figure 5B).

### Erythropoietin Ameliorated Contrast-Induced Nephrology-Associated Pyroptosis

**Figure 6:**
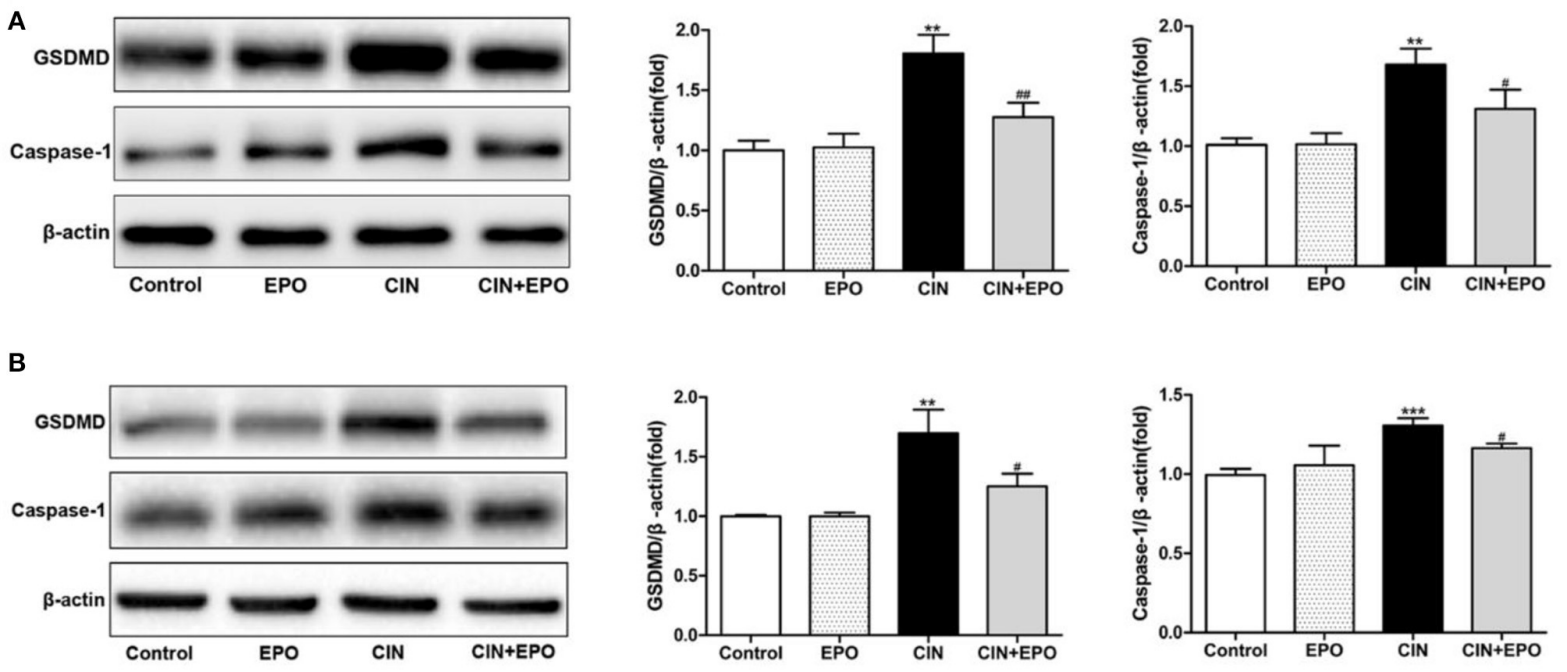
Effect of EPO on protein levels of GSDMD and caspase-1 in mice **(A)** and HK-2 cells **(B)**. Values are expressed as the mean ± standard deviation (*n* = 3). **P < 0.01, ***P < 0.001 vs. the CIN group. ^#^*P* < 0.05, ^*##*^*P* < 0.01 vs. the CIN group. EPO, erythropoietin; CIN, contrast-induced nephrology; GSDMD, gasdermin D.

Considering that pyroptosis may play a role in the development of CIN, we further explored the expression of GSDMD and caspase-1. After a 24-h stimulation with CM, the levels of GSDMD and caspase-1 of the CIN group were increased statistically than those of the control group. Notably, the presence of EPO can ameliorate cell pyroptosis of mouse suffering CIN (Figure 6A). The similar expression of GSDMD and caspase-1 was detected in a cell model (Figure 6B). Taken together, our data suggested that the EPO can attenuate pyroptosis in CIN.

### Erythropoietin May Protect Against Contrast-Induced Injury via the Janus Kinase/Signal Transducer and Activator of Transcription Signaling Pathway *in vitro* and *in vivo*

**Figure 7:**
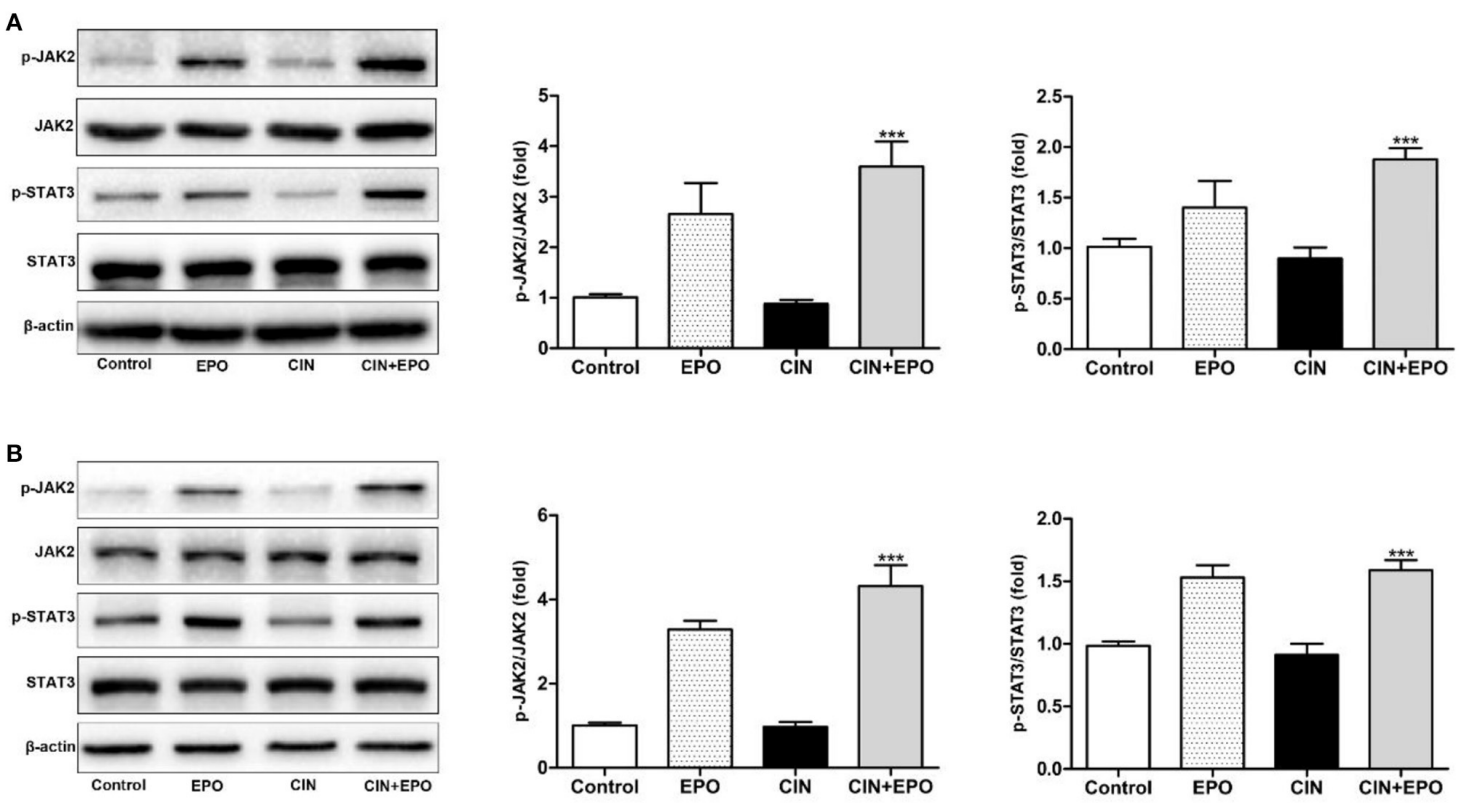
Effect of EPO on protein levels of the JAK2/STAT3 signaling pathway components induced by contrast in mice **(A)** and HK-2 cells **(B)**. Values are expressed as the mean ± standard deviation (*n* = 3). ***P < 0.001 vs. the CIN group. p-JAK2, phosphorylated Janus kinase 2; p-STAT3, phosphorylated signal transducer and activator of transcription 3; EPO, erythropoietin; CIN, contrast-induced nephrology.

Next, we explored the possible underlying mechanism associated with the regulatory effect of EPO on CIN. We measured the total and phosphorylated protein levels of JAK2 and STAT3 by WB in kidney tissue (Figure 7A). The results revealed that the expression of p-JAK2 and p-STAT3 was clearly enhanced at 24 h after EPO treatment. Furthermore, similar expression of these proteins was detected in the corresponding group of HK-2 cells (Figure 7B). In conclusion, JAK2/STAT3 signaling pathway activity played a role in alleviating CIN with EPO.

## Discussion

The emergence of CIN leads to increased mortality, prolonged hospitalization, and higher expenses (17). In addition to hydration, there is no special treatment widely used in the clinic to prevent CIN. Therefore, searching for effective treatments to prevent CIN and improve prognosis is crucial. EPO may protect multiple organs and tissues, such as the brain, heart, liver, lung, and kidney, and has been confirmed in experimental models or clinical trials (8, 9, 18–20). Meanwhile, studies did not report a higher risk of adverse events, such as symptomatic thrombosis, deep thrombophlebitis, myocardial infarction, stroke, and hypertension, which were caused by the EPO intervention (21–23). Otherwise, the use of EPO for the prevention and treatment of AKI in patients is still controversial. Prophylactic administration of EPO has a role in preventing AKI, reducing cardiac complications and lowering the incidence of prolonged vasopressor dependence in patients suffering cardiovascular surgery (7, 24, 25). Contrary to the former, a clinical trial concentrating on patients in intensive care units or undergoing complex cardiac surgery demonstrated that intravenous administration of EPO did not provide renal protection to patients with an increased risk of developing AKI (22, 23, 26). The timing of administration and risk stratification of patients may play a role in exerting EPO's renoprotection effect. Therefore, the controversy between different clinical trials requires more fundamental studies focused on the underlying mechanisms of EPO administration in AKI.

In our mouse and cell models of CIN, EPO pretreatment potently repaired histological injury of the kidney and reduced biochemical parameters of renal dysfunction. Meanwhile, EPO markedly alleviated apoptosis, shown as decreasing protein expression of Bax and cleaved caspase-3 and increasing level of Bcl-2. The presence of EPO also decreased the protein expression of GSDMD and caspase-1, which are involved in the occurrence of pyroptosis. Moreover, upregulation of JAK2 and STAT3 phosphorylation played a key role in these processes. These results suggested that EPO may attenuate CIN renal injury by suppressing apoptosis and pyroptosis, which depends on the JAK2/STAT3 signaling axis. To our knowledge, this is the first report documenting that EPO can ameliorate renal injury in experimental models of CIN through the JAK2/STAT3 signaling pathway.

At present, the pathogenesis involved in CIN has been researched widely. Several experimental studies have shown that the production of reactive oxygen species (ROS) or hypoxia-induced factor (HIF), markers of hypoxia, is enhanced after the administration of CM and then either directly or indirectly mediates cellular apoptosis and necrosis (27, 28). Quintavalle et al. revealed that increased ROS triggered the activation of Jun N-terminal kinases (JNK) and p38 stress kinases and then led to cell apoptosis (29). Our results suggested that CM leads to the apoptosis of renal tubular epithelial cells by enhancing the activation of caspase-3 and breaking the balance between Bcl-2 and Bax. GSDMD forms plasma membrane pores to cause pyroptotic cell death and the release of inflammatory factor in AKI (6). Tubular epithelial cell pyroptosis mediated by caspase-1 and caspase-11 has been confirmed in the process of CIN (16, 30). Our result is consistent with the previous study. Therefore, we concluded that cell apoptosis and pyroptosis play important roles in the process of CIN.

EPO renoprotection involves the attenuation of apoptosis, hypoxia, and inflammation and promotes microvascular cell survival and/or angiogenesis (31, 32). Moreover, EPO reduced macrophage infiltration and enhanced the phenotypic switch toward M2 macrophages in an experimental model of rhabdomyolysis-induced AKI (11, 33). Nevertheless, the relevant processes at the cellular and molecular levels were not completely illustrated in CIN. Previous studies have proven that EPO can prevent renal dysfunction caused by CM, alleviate renal morphologic tubular injuries, and decrease apoptosis by activating the JAK2/STAT5 signaling pathway (12). Consistent with previous discoveries, caspase-3, Bcl-2, and Bax were involved in the apoptosis process in CIN. Meanwhile, pathological and functional injury was improved by EPO. In our study, CM could directly induce cytotoxic effects, while EPO could reactivate cells. Indeed, we also found that EPO alleviated the renal injury caused by CM in a concentration- and time-dependent manner.

A mounting body of evidence has revealed the involvement of the JAK/STAT signaling pathway in various diseases (10, 13, 20, 34). To the best of our knowledge, no studies have investigated the role of JAK2/STAT3 signaling in CIN. It was reported that the phosphorylated activation of JAK2/STAT3 played a part in the induction of apoptosis, autophagy, inflammation, and oxidative stress and that the activation of the JAK2-STAT3 signaling pathway protected tissue against acute injury in some studies (35–37). In contrast, the renoprotective effect of curcumin in AKI caused by lipopolysaccharide (LPS) or severe acute pancreatitis may be associated with inflammation reduction mediated by suppression of JAK2/STAT3 signaling pathway (38, 39). In our study, increased phosphorylation of JAK2/STAT3 after EPO treatment alleviated CIN by ameliorating cell apoptosis and pyroptosis *in vivo* and *in vitro*. In the future, the effects of EPO on apoptosis and pyroptosis should be explored further to distinguish more treatment targets and promote the clinical application of EPO for CIN.

## Conclusion

In conclusion, the findings of this study suggest that EPO protects the kidney against histological injury and reduces biochemical parameters of renal dysfunction in CIN. Concerning the underlying mechanism, the findings indicate that EPO ameliorated apoptosis and pyroptosis via activation of the JAK2/STAT3 signaling pathway. The findings of this study are promising, and they need to be confirmed in human patients to explore the possibility of using EPO as a therapeutic agent for CIN.

## Data Availability Statement

The raw data supporting the conclusions of this article will be made available by the authors, without undue reservation.

## Ethics Statement

The animal study was reviewed and approved by the Animal Care and Use Committee of the West China Hospital of Sichuan, China.

## Author Contributions

JY and XW performed the experiments. JY was a major contributor to the writing of the manuscript. JZ and LY were responsible for the experimental design and statistical analysis. LJ, SW, and XC participated in the experiments and data analysis. All the authors have read and approved the final version of the manuscript.

## Conflict of Interest

The authors declare that the research was conducted in the absence of any commercial or financial relationships that could be construed as a potential conflict of interest.
