# Supplementary figures and images for: Erythropoietin Attenuates Experimental Contrast-Induced Nephrology: A Role for the Janus Kinase 2/Signal Transducer and Activator of Transcription 3 Signaling Pathway
Source: Front Med (Lausanne). 2021 Apr 13;8:634882. doi: 10.3389/fmed.2021.634882 (PMC8076515; doi:10.3389/fmed.2021.634882)

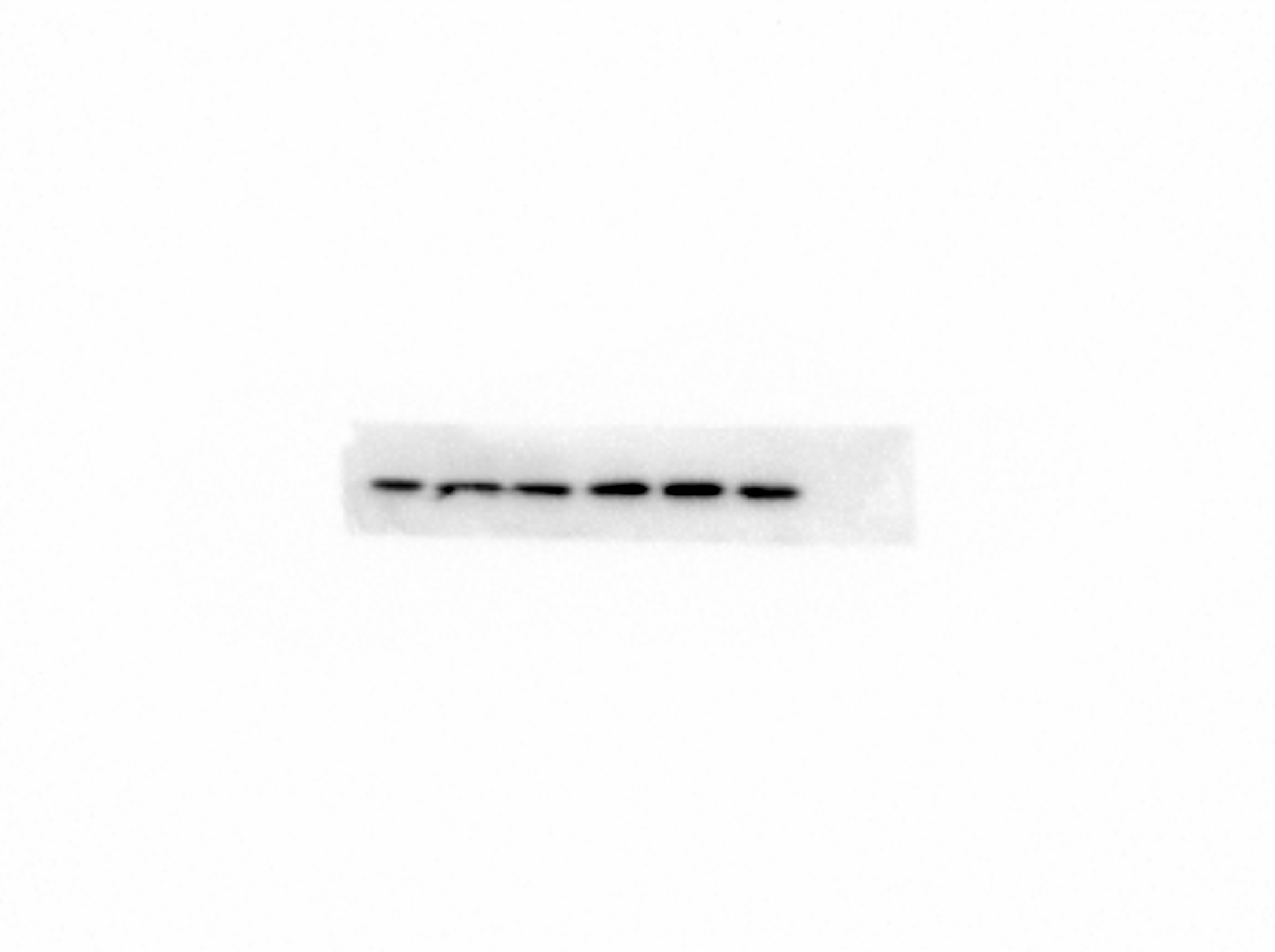

Supplement: Supplementary file 1 [file Data_Sheet_1.ZIP › WB╘¡═╝/fig.3-(Bax).tif]

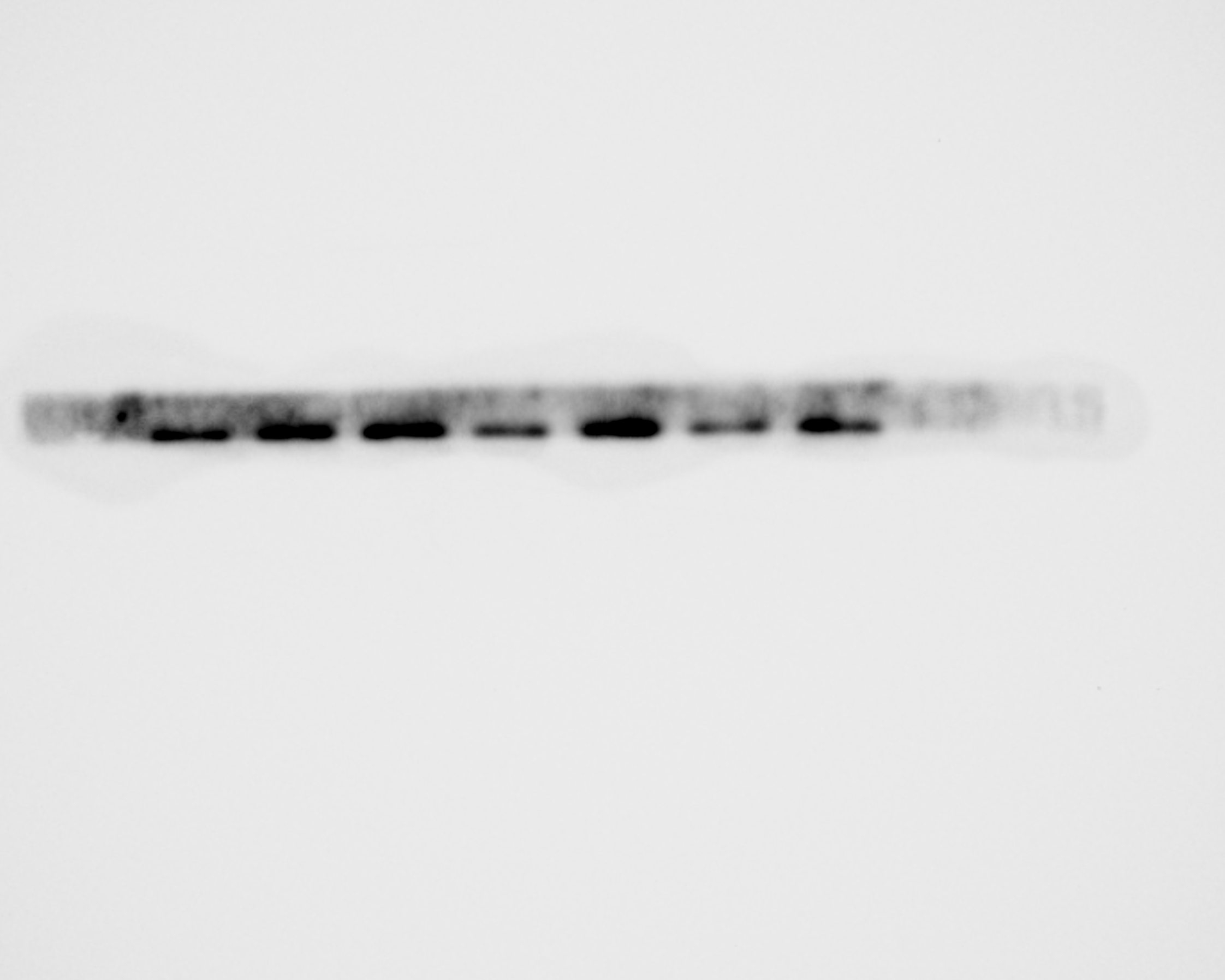

Supplement: Supplementary file 1 [file Data_Sheet_1.ZIP › WB╘¡═╝/fig.3-(Bcl-2).tif]

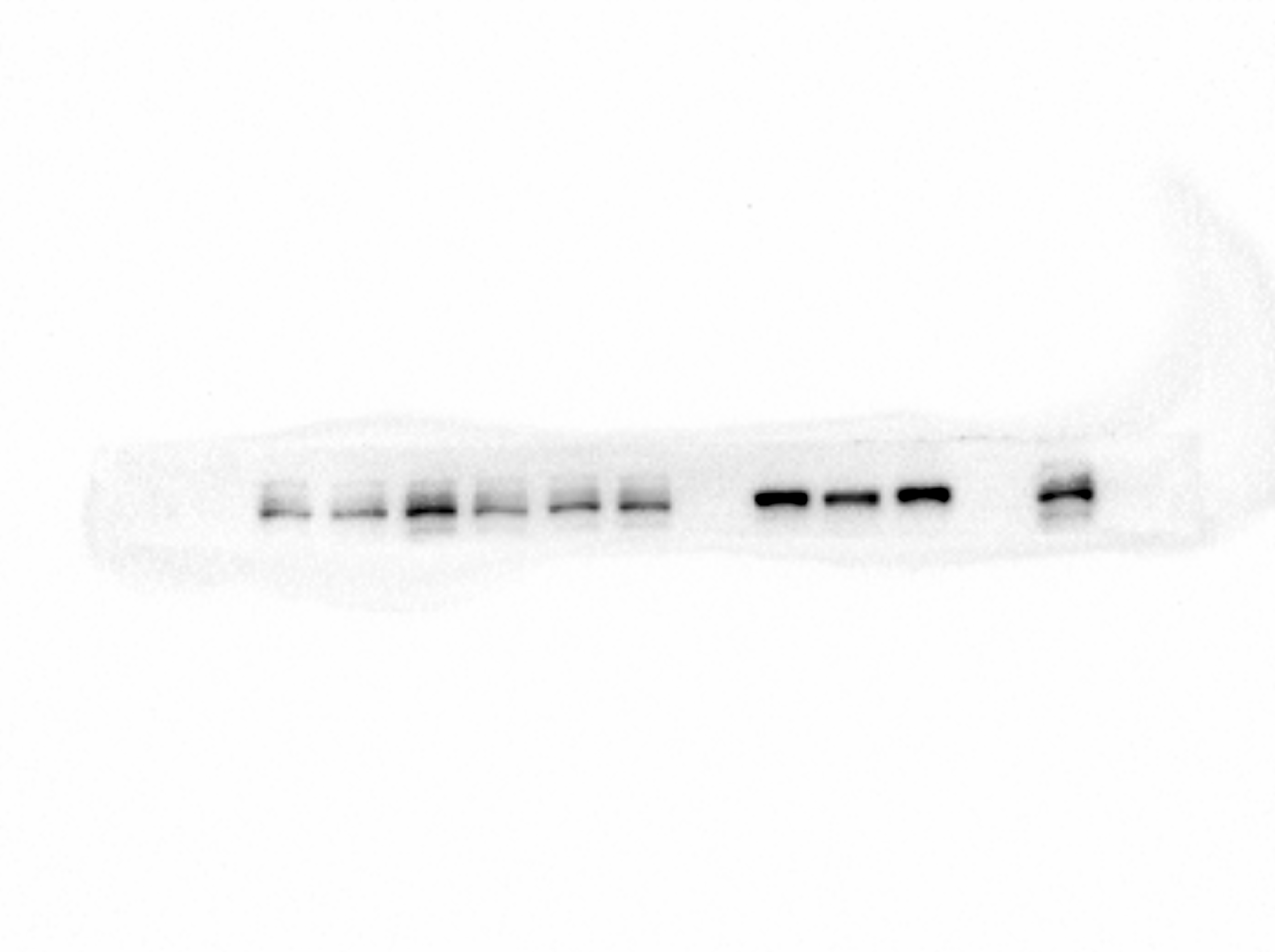

Supplement: Supplementary file 1 [file Data_Sheet_1.ZIP › WB╘¡═╝/fig.3-(c-caspase3).tif]

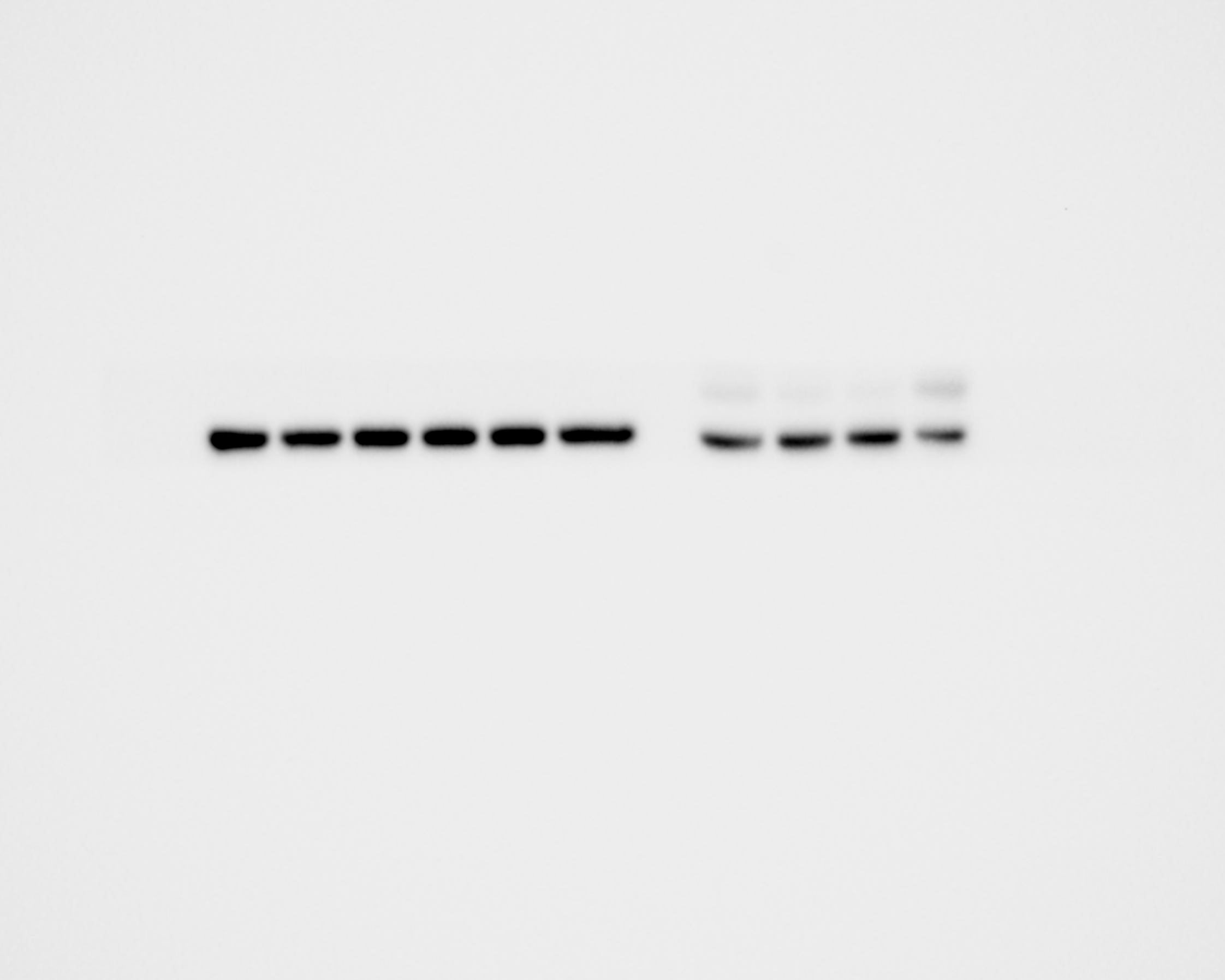

Supplement: Supplementary file 1 [file Data_Sheet_1.ZIP › WB╘¡═╝/fig.3-(a┬-actin).tif]

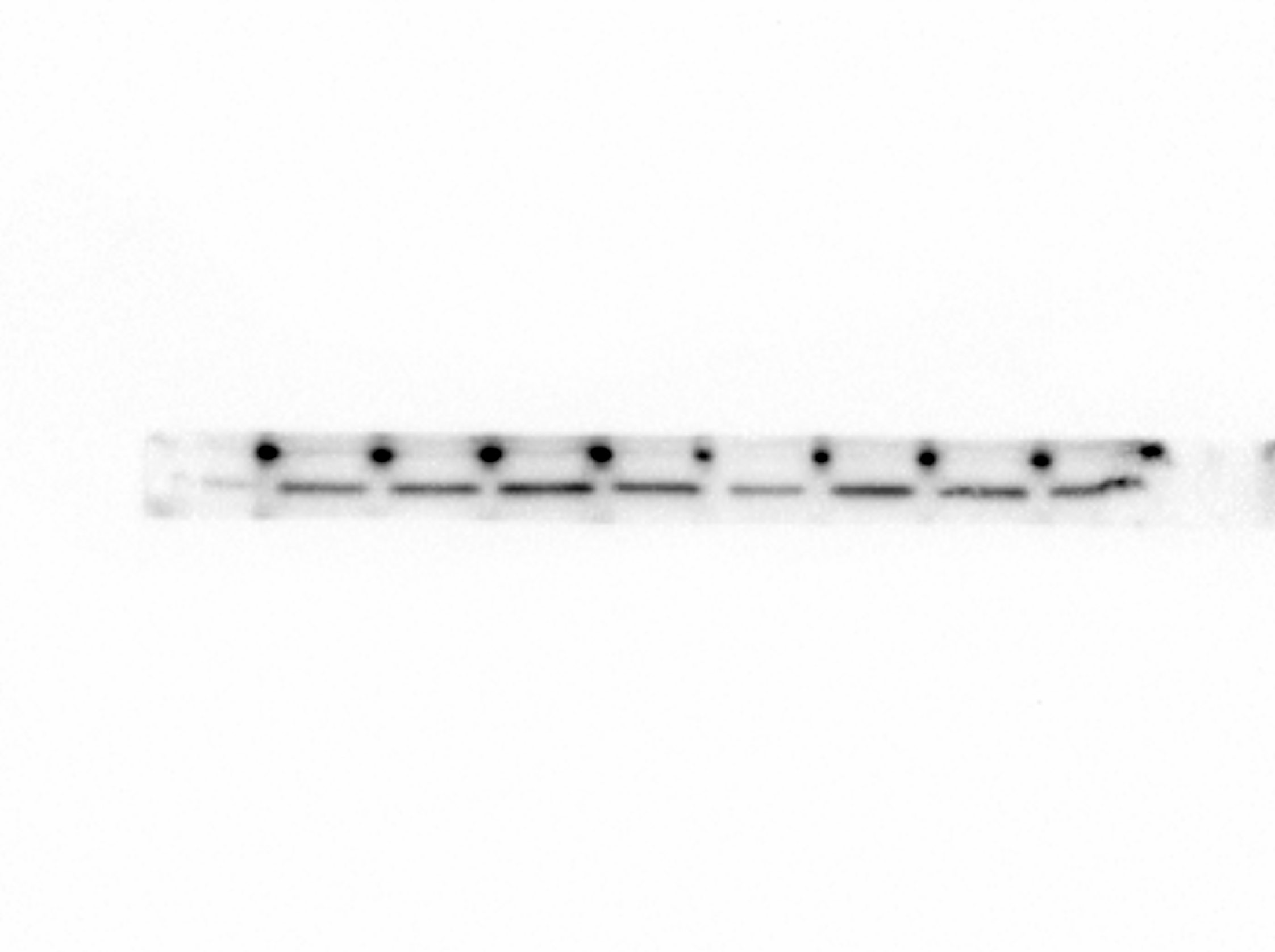

Supplement: Supplementary file 1 [file Data_Sheet_1.ZIP › WB╘¡═╝/fig.4-(Bax).tif]

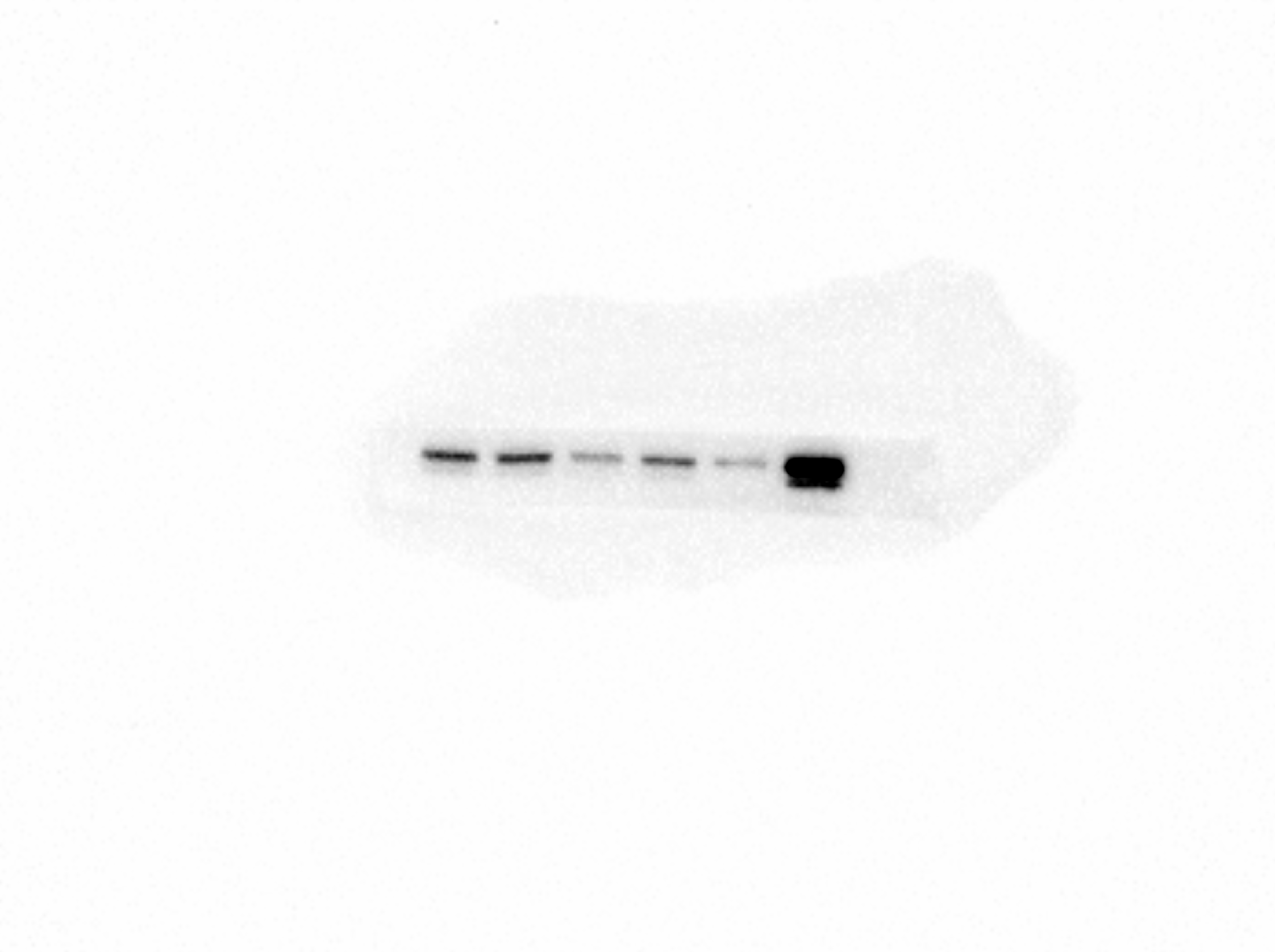

Supplement: Supplementary file 1 [file Data_Sheet_1.ZIP › WB╘¡═╝/fig.4-(Bcl-2).tif]

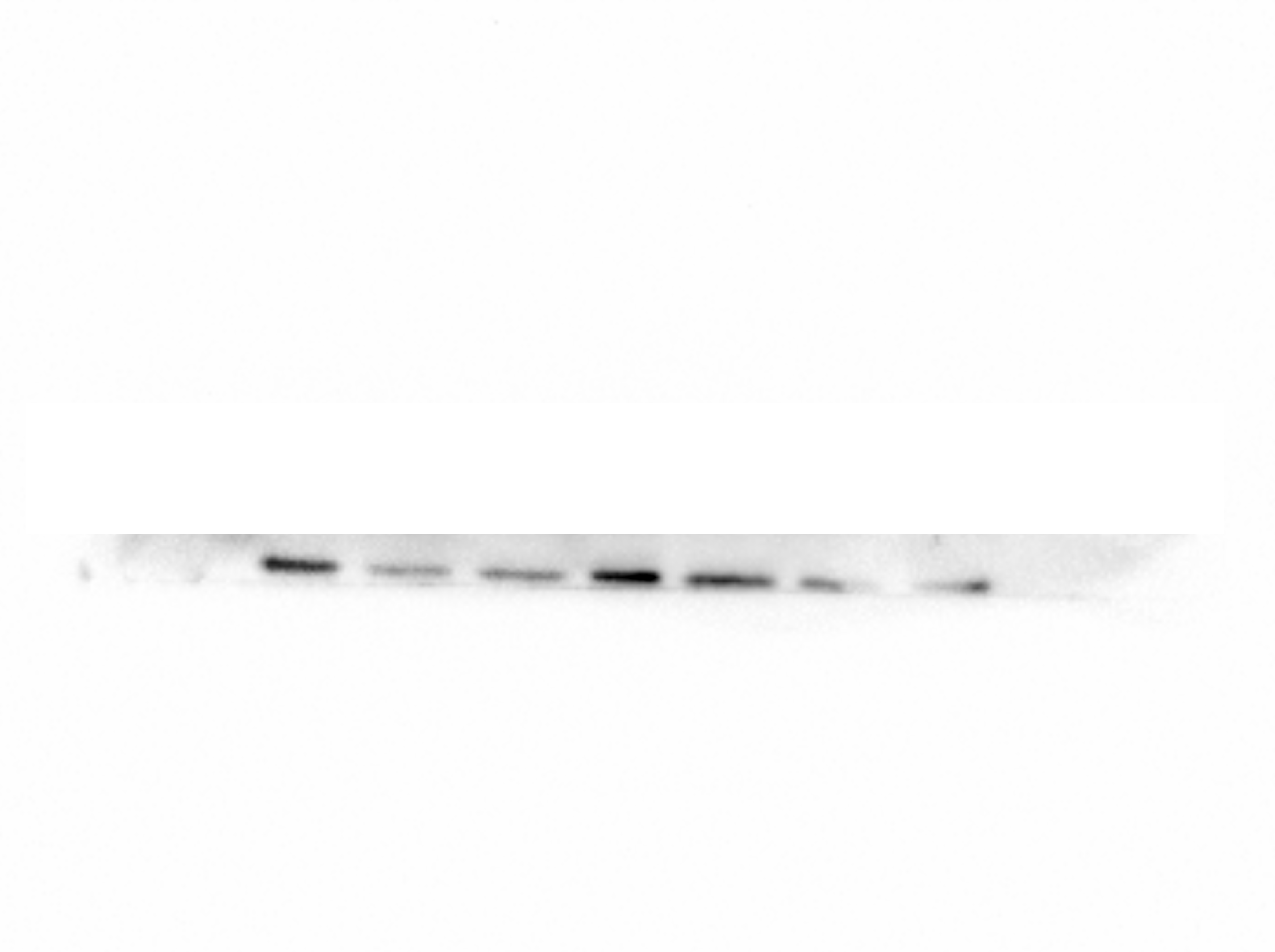

Supplement: Supplementary file 1 [file Data_Sheet_1.ZIP › WB╘¡═╝/fig.4-(c-caspase3).tif]

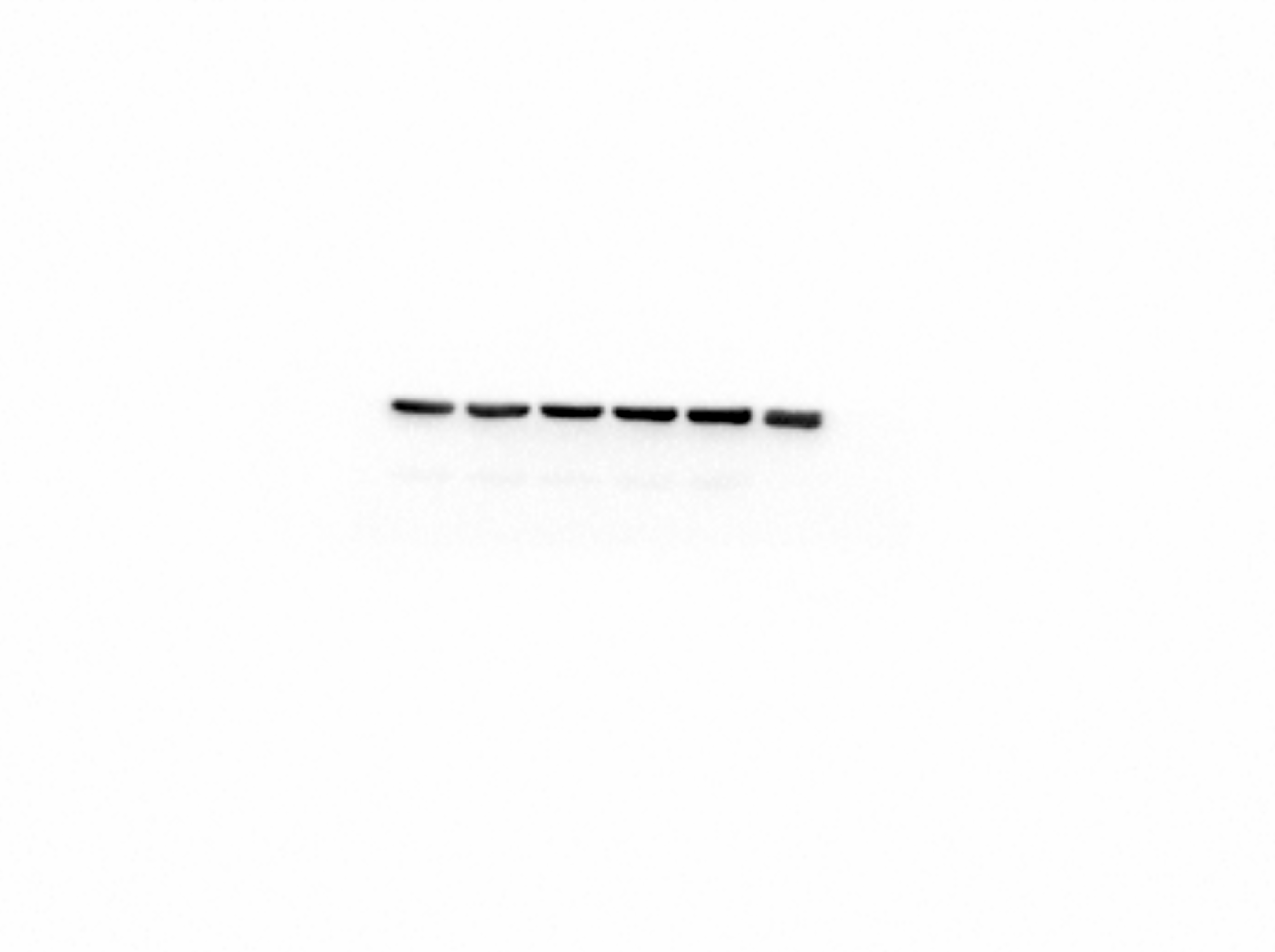

Supplement: Supplementary file 1 [file Data_Sheet_1.ZIP › WB╘¡═╝/fig.4-(a┬-actin).tif]

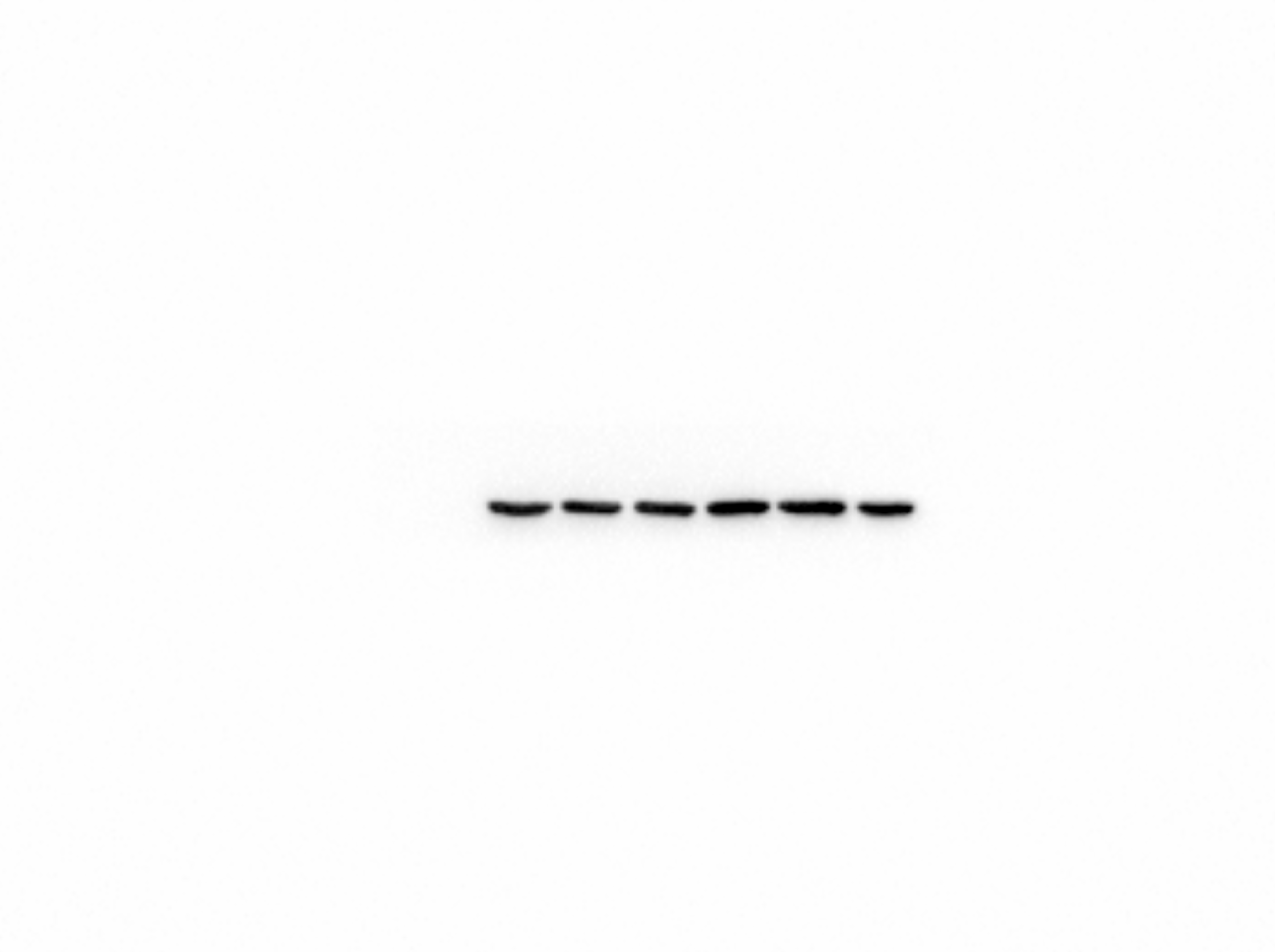

Supplement: Supplementary file 1 [file Data_Sheet_1.ZIP › WB╘¡═╝/fig.6A-(JAK2).tif]

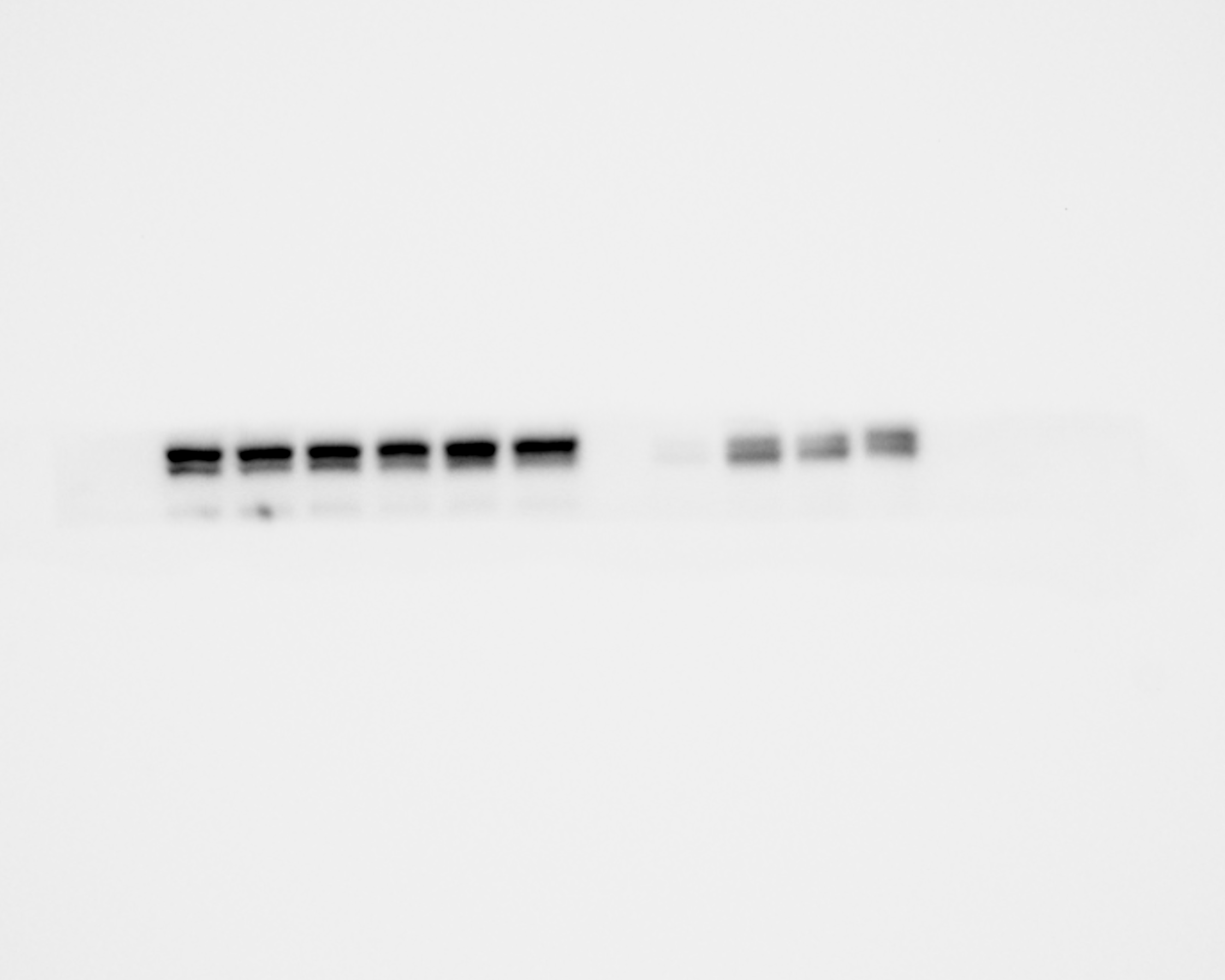

Supplement: Supplementary file 1 [file Data_Sheet_1.ZIP › WB╘¡═╝/fig.6A-(STAT3).tif]

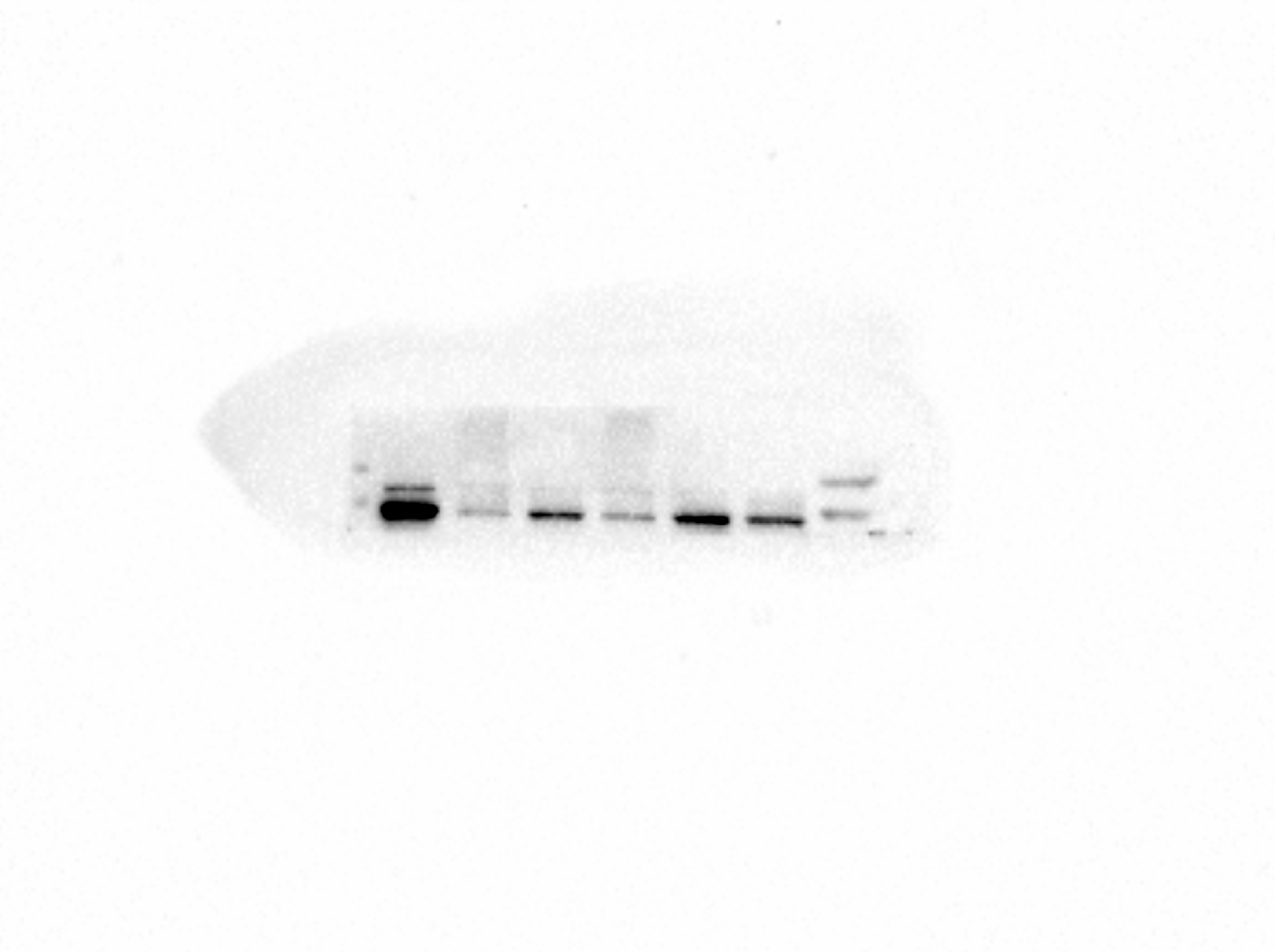

Supplement: Supplementary file 1 [file Data_Sheet_1.ZIP › WB╘¡═╝/fig.6A-(p-JAK2).tif]

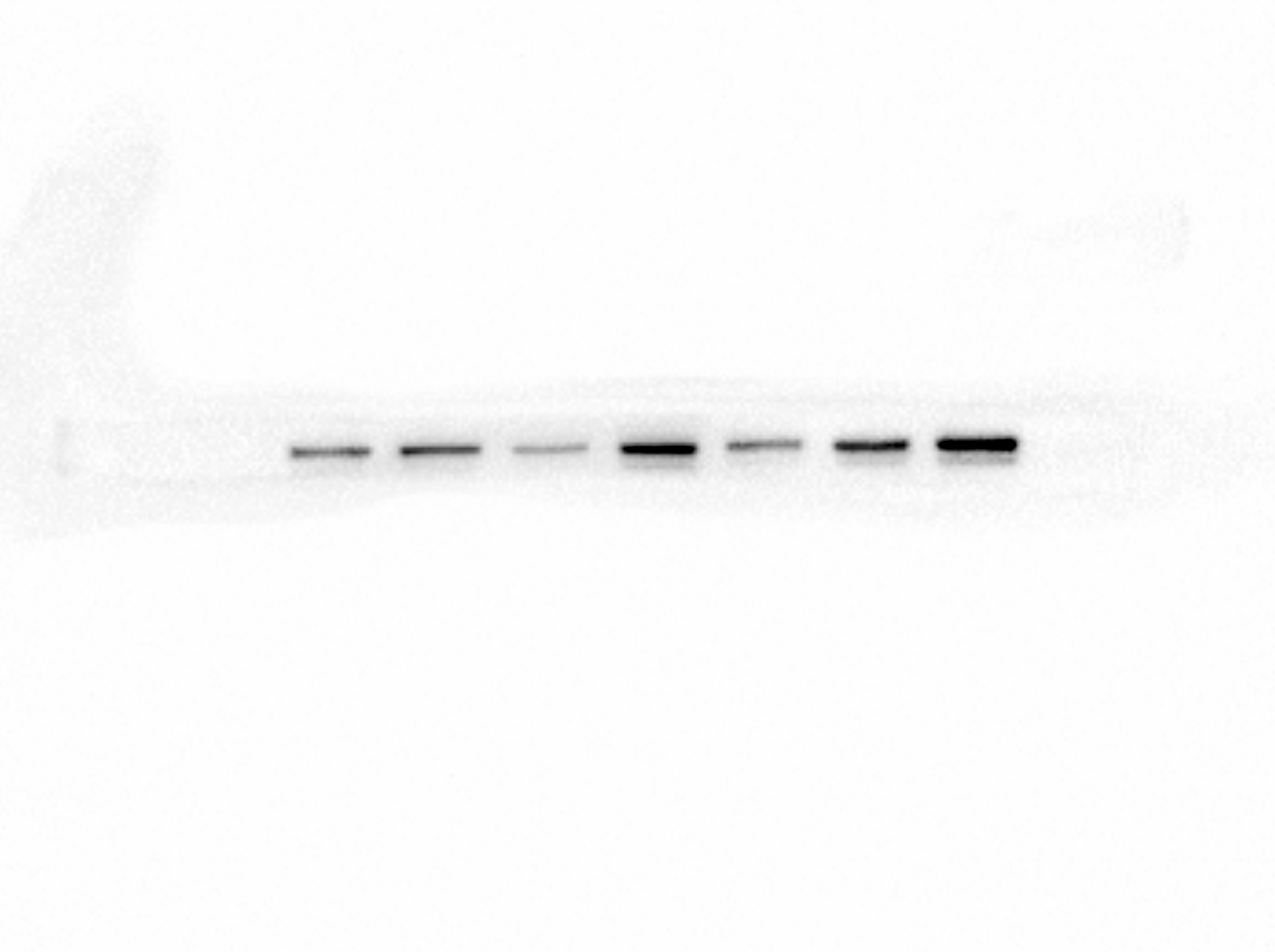

Supplement: Supplementary file 1 [file Data_Sheet_1.ZIP › WB╘¡═╝/fig.6A-(p-STAT3).tif]

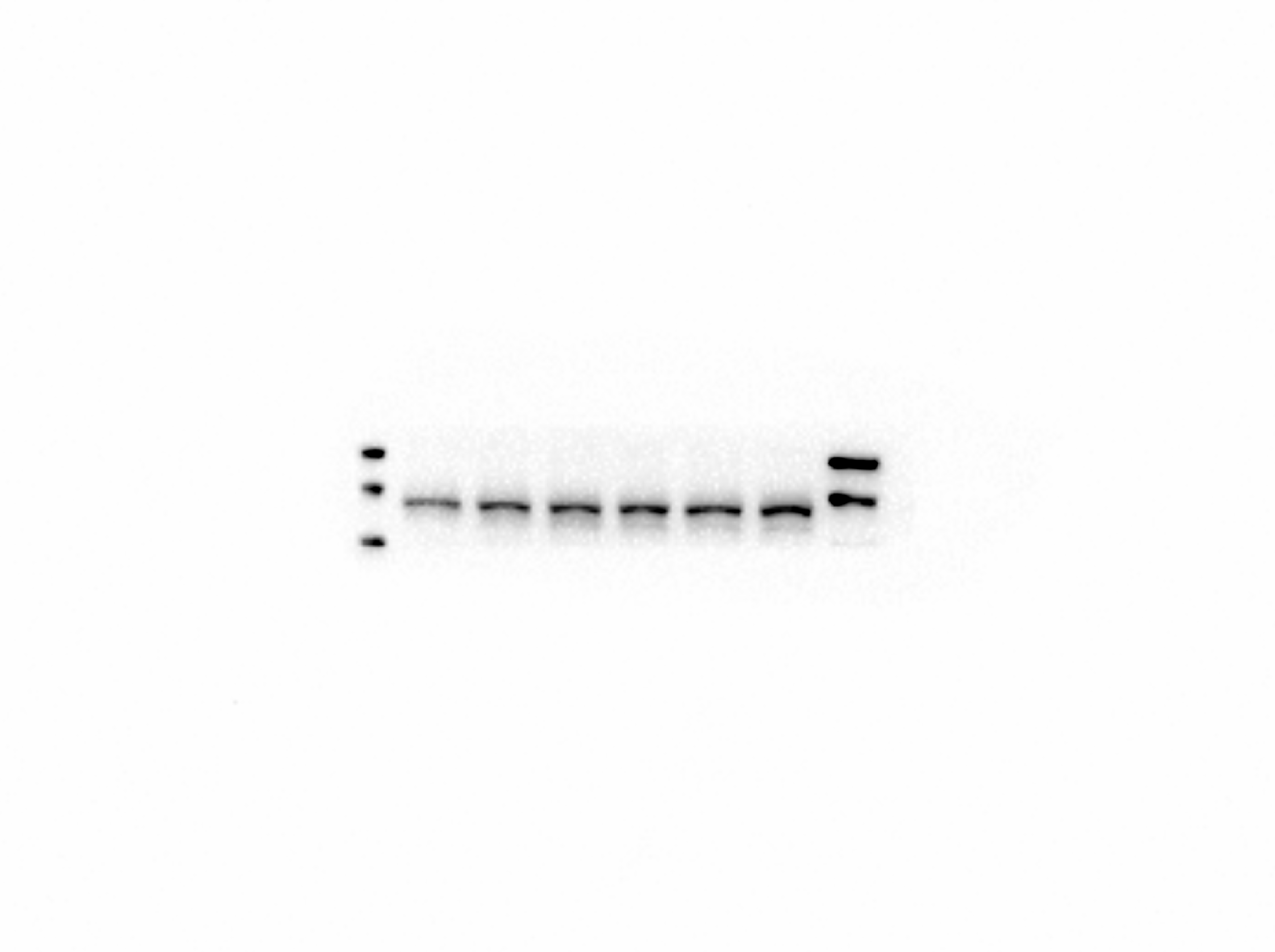

Supplement: Supplementary file 1 [file Data_Sheet_1.ZIP › WB╘¡═╝/fig.6B-(JAK2).tif]

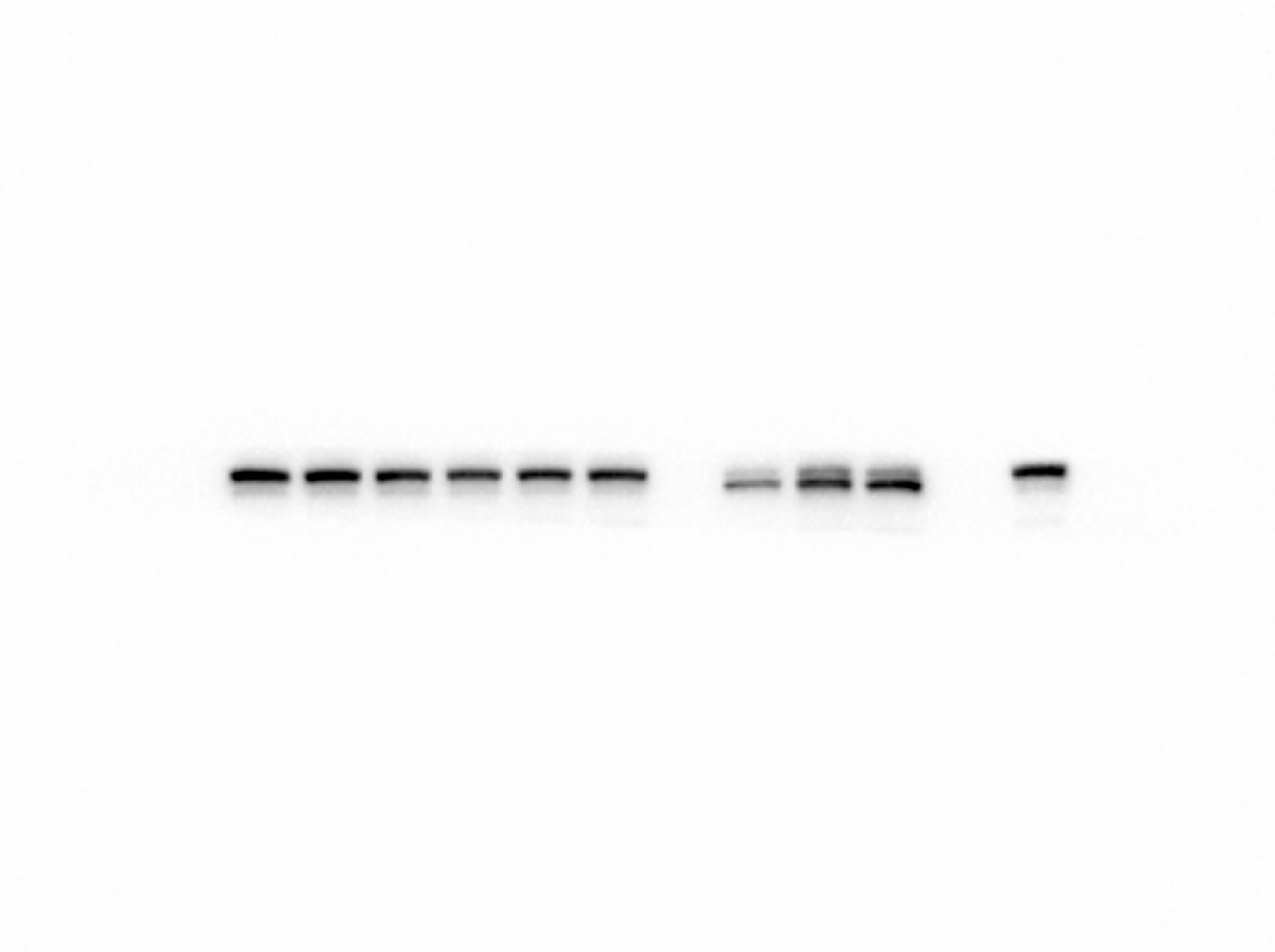

Supplement: Supplementary file 1 [file Data_Sheet_1.ZIP › WB╘¡═╝/fig.6B-(STAT3).tif]

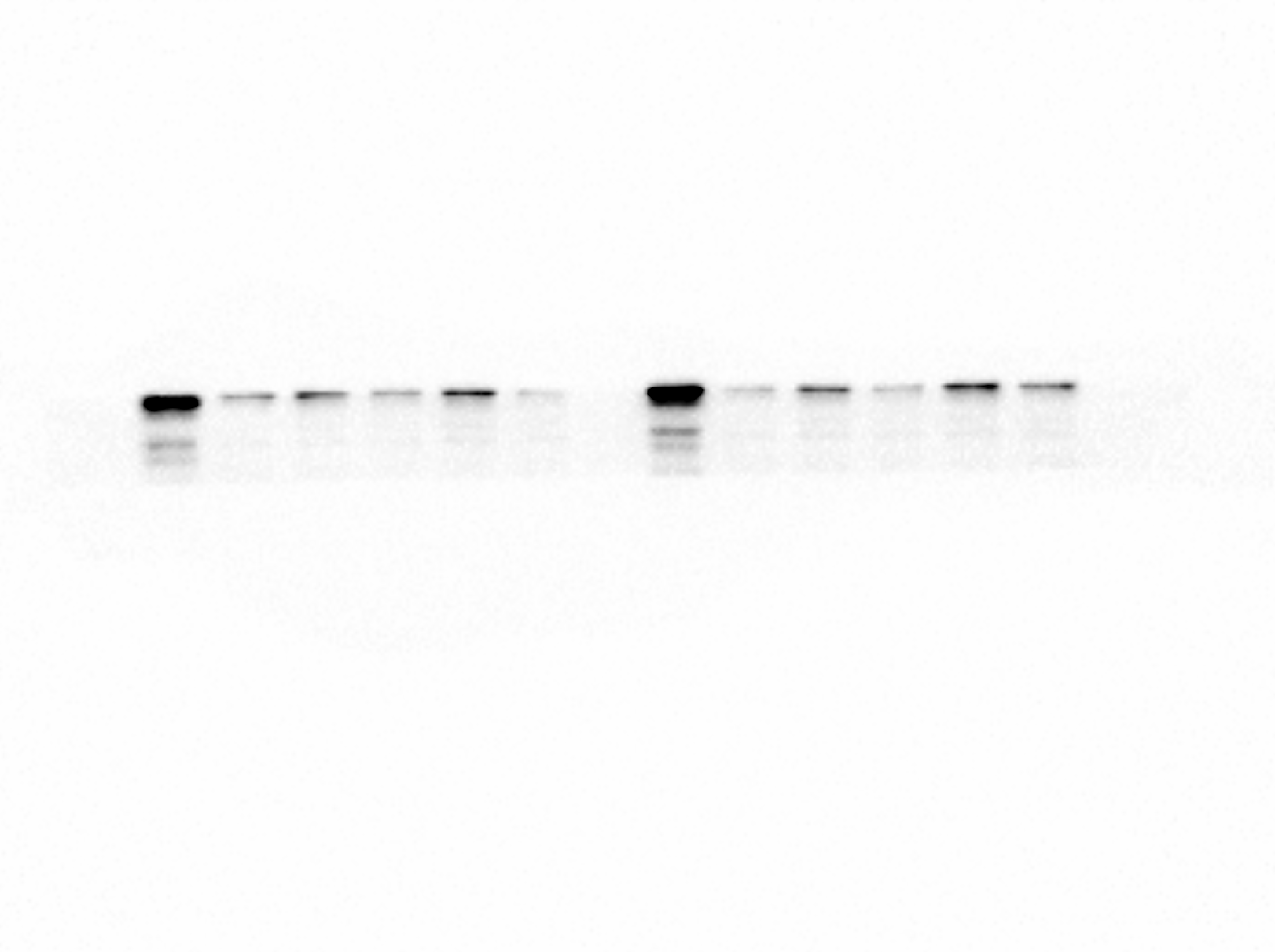

Supplement: Supplementary file 1 [file Data_Sheet_1.ZIP › WB╘¡═╝/fig.6B-(p-JAK2).tif]

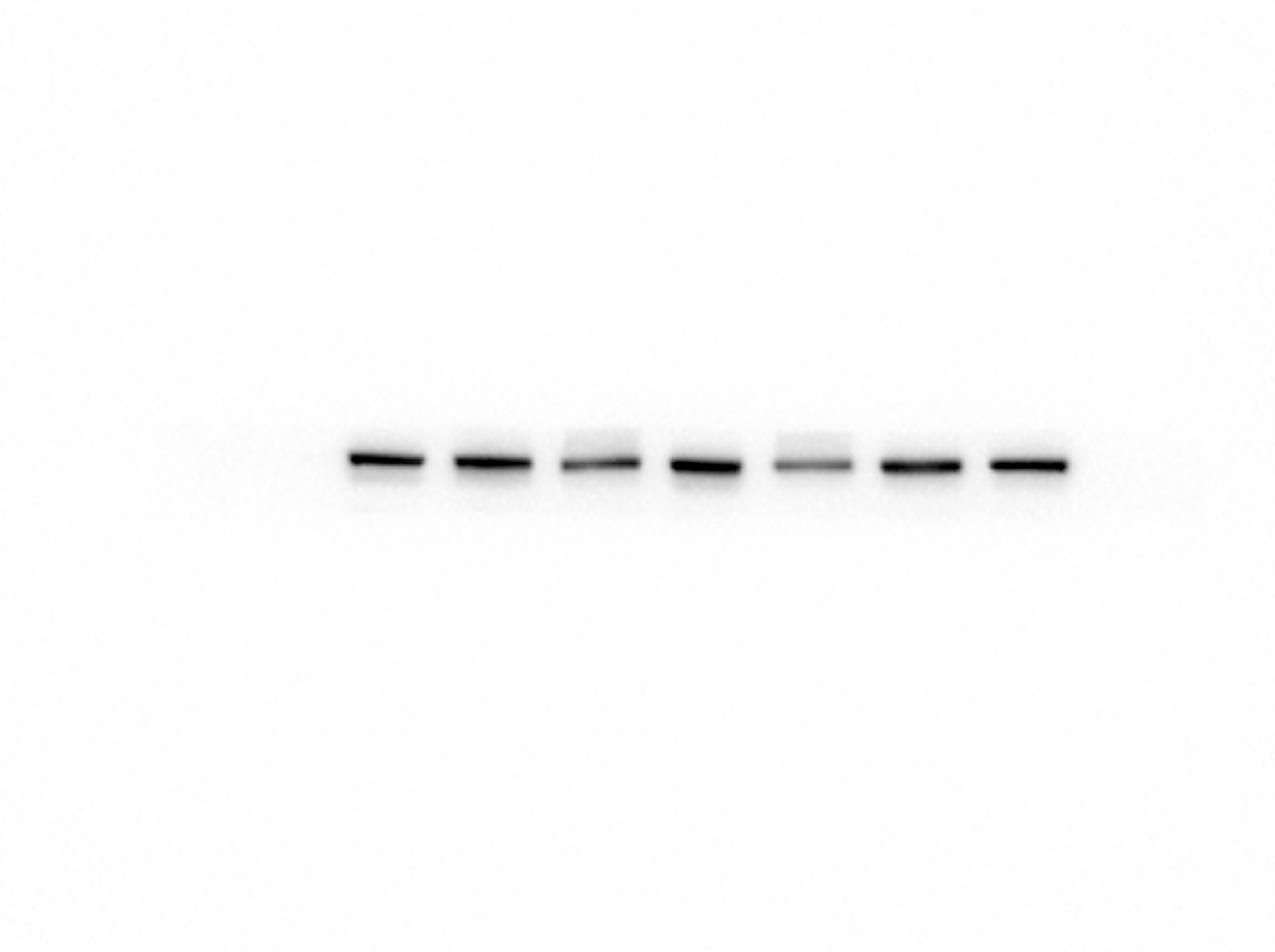

Supplement: Supplementary file 1 [file Data_Sheet_1.ZIP › WB╘¡═╝/fig.6B-(p-STAT3).tif]
